# Principles of General Physiology

**Published:** 1915-12

**Authors:** 


**Principles of General Physiology.** By Wm. Bayliss. Longmans, Green & Co. Price 21s.

This book is somewhat off the usual lines. It is not intended to be a text-book for candidates for examinations, nor does it touch upon the experimental details of physiological research. It rather aims to bring into line the cognate sciences, and to introduce the reader to the relationship between physiological facts and such phenomena as adsorption, enzyme action, osmosis, surface action, the colloidal state, electrolytes, and properties of the cell. There is, however, a chapter on each of the main physiological functions—respiration, reflex action, the eye, hormones, etc. It is not exactly a text-book of comparative physiology, in the sense that it does not devote special attention to the functions of the lower forms of animal life.

The author brings to his task a very wide acquaintance with those parts of physics, biology and chemistry which throw a light upon physiology. The style, considering the complexity of many of the subjects, is clear. The book is well up to date. and eminently scientific. Amongst the illustrations are photographs of a number of the famous investigators whose researches are incorporated in the text.

